# Supplementary material for: Splice-Junction-Based Mapping of Alternative Isoforms in the Human Proteome
Source: Cell Rep. Author manuscript; Available in PMC 2020 Jan 15. (PMC6961840; doi:10.1016/j.celrep.2019.11.026)
Supplement: 2 [file NIHMS1546469-supplement-2.zip › DF1/PXD006675/Aorta/Aorta_4_NEXN_AEQVNEDEENQDTAK.pdf]

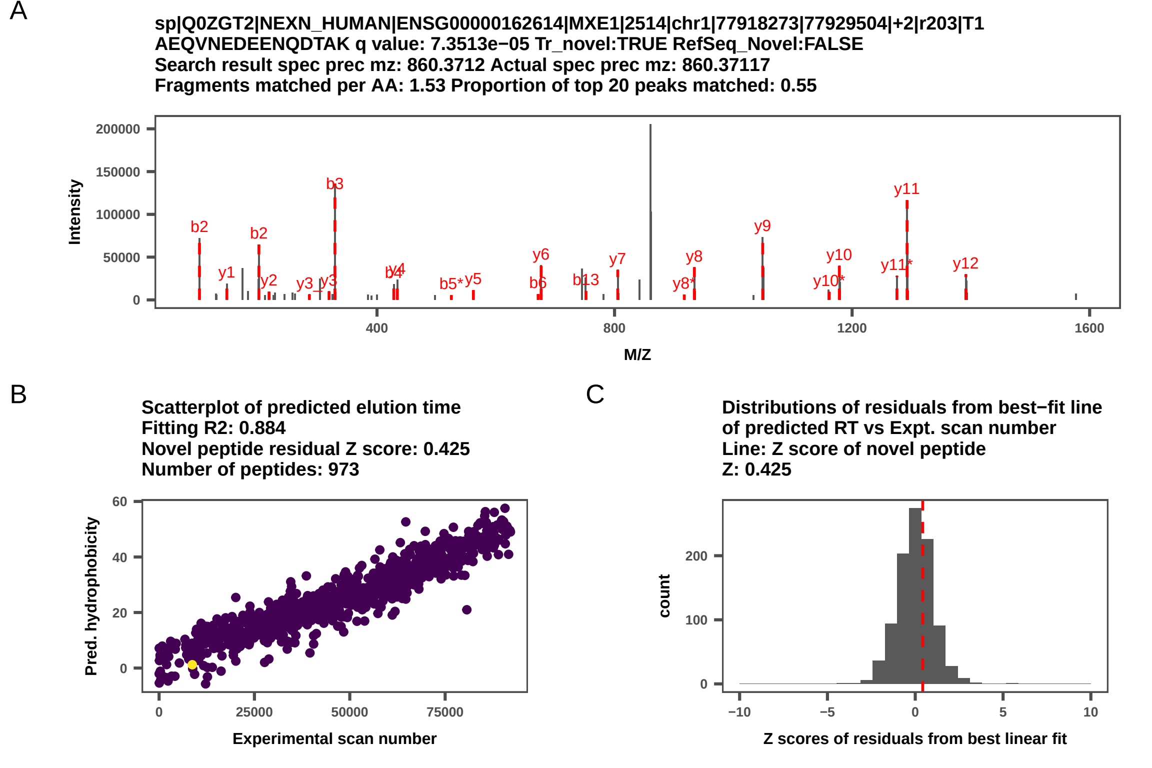
A
sp|Q0ZGT2|NEXN_HUMAN|ENSG00000162614|MXE1|2514|chr1|77918273|77929504|+2|r203|T1
AEQVNEDEENQDTAK q value: 7.3513e−05 Tr_novel:TRUE RefSeq_Novel:FALSE
Search result spec prec mz: 860.3712 Actual spec prec mz: 860.37117
Fragments matched per AA: 1.53 Proportion of top 20 peaks matched: 0.55
Intensity
M/Z
b2
y1
b2
y2
y3_y3
b3
b4
y4
b5*
y5
y6
b6
b13
y7
y8*
y8
y9
y10*
y10
y11*
y11
y12
B
Scatterplot of predicted elution time
Fitting R2: 0.884
Novel peptide residual Z score: 0.425
Number of peptides: 973
Pred. hydrophobicity
Experimental scan number
C
Distributions of residuals from best−fit line
of predicted RT vs Expt. scan number
Line: Z score of novel peptide
Z: 0.425
count
Z scores of residuals from best linear fit
